# The Influence of Face Gaze by Physicians on Patient Trust: an Observational Study

**DOI:** 10.1007/s11606-021-06906-2

**Published:** 2021-05-24

**Authors:** Chiara Jongerius, Jos W. R. Twisk, Johannes A. Romijn, Timothy Callemein, Toon Goedemé, Ellen M. A. Smets, Marij A. Hillen

**Affiliations:** 1 Department of Medical Psychology, Amsterdam Public Health, Amsterdam UMC, University of Amsterdam, Amsterdam, The Netherlands; 2 Department of Epidemiology & Data Science, Amsterdam UMC, VU University, Amsterdam, The Netherlands; 3 Department of Medicine, Amsterdam UMC, University of Amsterdam, Amsterdam, The Netherlands; 4 PSI-EAVISE, Electrical Engineering Technology (ESAT), KU Leuven, De Nayer Campus Sint-Katelijne-Waver, Sint-Katelijne-Waver, Belgium

**Keywords:** face gaze, patient trust, physician empathy, eye-tracking, social anxiety

## Abstract

**Background:**

Physicians’ gaze towards their patients may affect patients’ trust in them. This is especially relevant considering recent developments, including the increasing use of Electronic Health Records, which affect physicians’ gaze behavior. Moreover, socially anxious patients’ trust in particular may be affected by the gaze of the physician.

**Objective:**

We aimed to evaluate if physicians’ gaze towards the face of their patient influenced patient trust and to assess if this relation was stronger for socially anxious patients. We furthermore explored the relation between physicians’ gaze and patients’ perception of physician empathy and patients’ distress.

**Design:**

This was an observational study using eye-tracking glasses and questionnaires.

**Participants:**

One hundred patients and 16 residents, who had not met before, participated at an internal medicine out-patient clinic.

**Measures:**

Physicians wore eye-tracking glasses during medical consultations to assess their gaze towards patients’ faces. Questionnaires were used to assess patient outcomes. Multilevel analyses were conducted to assess the relation between physicians’ relative face gaze time and trust, while correcting for patient background characteristics, and including social anxiety as a moderator. Analyses were then repeated with perceived empathy and distress as outcomes.

**Results:**

More face gaze towards patients was associated with lower trust, after correction for gender, age, education level, presence of caregivers, and social anxiety (*β*=−0.17, *P*=0.048). There was no moderation effect of social anxiety nor a relation between face gaze and perceived empathy or distress.

**Conclusions:**

These results challenge the notion that more physician gaze is by definition beneficial for the physician-patient relationship. For example, the extent of conversation about emotional issues might explain our findings, where more emotional talk could be associated with more intense gazing and feelings of discomfort in the patient. To better understand the relation between physician gaze and patient outcomes, future studies should assess bidirectional face gaze during consultations.

## INTRODUCTION

Gaze is a crucial aspect of communication.^1, 2^ Gaze towards the eyes or face has previously been used as a proxy for “eye contact”.^2^ It transmits social and attentional information and can direct a conversation.^1, 3, 4^ Physicians’ gaze is therefore an essential aspect of physician-patient communication. Gaze between physician and patient affects the patient during the consultation.^5–7^ For instance, during medical consultations, patients follow the physicians’ gaze towards the computer screen.^7^ Physician gaze has also been related to outcomes after the consultation such as patients’ medication adherence and their physical and cognitive functioning.^5, 6, 8, 9^

Sub-optimal levels of gaze between physicians and patients may have negative effects on physician-patient relationships,^10–12^ including reduced trust of patients in their physicians. This is especially relevant considering present-day characteristics of the consultation, such as the increasing use of Electronic Health Records, which may reduce the physicians’ amount of gaze towards the patient.^13–16^ If increased use of Electronic Health Records leads to reduced gaze towards the patients, this may eventually harm patients’ trust in their physician, whereas trust is crucial for the quality of the patient-physician relationship.^17^ Ultimately, reduced trust is suggested to lead to harmful long-term effects, such as less medication adherence and lower patient well-being.^17–20^

The amount of physician gaze towards their patients may not only affect trust, but also patients’ perception of physician empathy^21^ and their emotional wellbeing, particularly distress.^22^ Therefore, it is worthwhile to investigate the gaze of the physician and its implications for patients.

Physicians’ gaze may affect patients differently since individual differences, especially social anxiety, influence the perception of gaze towards the eyes.^23–25^ Individuals who suffer from social anxiety tend to feel unease in social interactions and may therefore experience gaze as unpleasant.^26^ Social anxiety ranks the third most common mental disorder (after depression and substance abuse) and is associated with greater health care utilization and lower health-related quality of life.^27, 28^ Due to gaze aversion, these patients may need a different communication approach than others. Whereas higher levels of physician gaze are commonly considered beneficial to the physician-patient relation,^11^ more gaze could induce distress and negatively affect trust among socially anxious patients. Despite its apparent relevance, we do not yet know whether the negative experience of physician gaze for socially anxious patients can be extrapolated to the medical setting.

We aimed to evaluate whether the level of physician gaze towards their patient predicts patients’ trust. We additionally aimed to explore the relation between physician face gaze and patients’ perception of physician empathy, and their distress. Furthermore, we assessed whether social anxiety moderates the association between physicians’ gaze and patients’ trust, perceived level of empathy, and distress. The results of our study can be used to support physicians in optimizing their nonverbal communication behaviors.

## METHODS

### Design and Procedure

**Figure 1:**
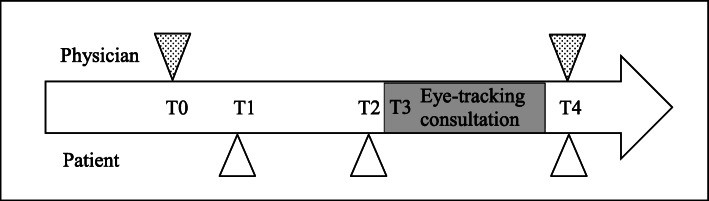
Graphic representation of the study measurement points. *Note:* The triangles symbolize the administration of questionnaires. T0 was at an unstructured time point convenient to the physician. T1 occurred around two weeks before the consultation. T2 occurred around 10 minutes before the consultation. T4 was as soon as the patient left the consultation room.

This was an observational study using eye-tracking to assess gaze patterns of physicians and validated questionnaires to assess patient outcomes. The study was conducted at the outpatient clinic of an internal medicine department of a Dutch hospital. It was judged to be exempt from approval by the ethical committee (protocol number W17_107). The study encompassed measurements at 5 time points (T0-T4) (see Fig. 1). At T0, physicians were recruited using snowballing sampling. To limit physicians’ awareness of the purpose of the study, physicians were informed that their use of the Electronic Health Record was the subject of research.

Physicians signed informed consent and completed a baseline questionnaire. Two weeks before visiting for their follow-up consultation, patients—who did not meet the physician before—were telephonically invited to participate. Upon agreement, patients signed informed consent and completed a baseline questionnaire (T1). Just prior to the consultation, patients indicated their current level of distress in a questionnaire (T2). The consultation was video-recorded with a camera positioned in a corner of the consultation room, and the gaze of the physician was tracked using mobile eye-tracking glasses (T3). Immediately after the consultation, physicians and patients responded to questionnaires assessing study outcomes (T4). Patients received a 15 euro gift card for participation. Data were collected between February 2018 and May 2019.

### Participants

Participating physicians were residents of internal medicine. Participating patients visited the outpatient clinic for a scheduled follow-up consultation. Patients were eligible who had had no previous consultations with the physician, spoke Dutch fluently, were older than 18 years, and had no mental disabilities or other serious cognitive impairments that could hinder their study participation.

### Instruments

#### Eye-Tracking Glasses

The gaze of the physician towards the patient’s face was tracked using Tobii Pro Glasses 2^29^ (T3). The eye-tracking glasses are equipped with two cameras per eye (measuring pupil movement) and a camera that records the environment. The included accelerometer and gyroscope sensors enable the software to differentiate between head and eye movements, limiting the influence of head movements on eye-tracking data. Calibration was performed for each physician to ensure measurement accuracy, using specific software.^29–31^ The face of the patient comprised the so-called area-of-interest, which is used to assess the dwell time (the duration of gaze in a specific “area-of-interest”).^32^

#### Questionnaires

Questionnaires were provided on paper or electronically (using Qualtrics.com), depending on the participant’s preference.^33^ Sociodemographic characteristics assessed were age, gender, and nationality of both physicians and patients (T0 and T1).

Patients’ trust in their physician was assessed using the Wake Forest Physician Trust Scale (WFPTS) consisting of 10 items (1= “totally disagree”–5=“totally agree) (T4).^34, 35^ This scale is used to assess patients’ interpersonal trust in their individual health care provider. Higher scores indicate higher levels of trust. The reliability of the scale in our sample was high (Cronbach’s alpha=.82).

The patient’s perceived level of physician empathy was measured with the Consultation And Relational Empathy (CARE), a 10-item scale (1= “poor”–5=“excellent” and an additional option “does not apply”) (T4).^36, 37^ This scale measures relational empathy in the clinical context, which is the ability to (1) understand the patient’s situation, perspective, and feelings; (2) communicate that understanding and checking its accuracy; and (3) to act on that understanding in a helpful way. Higher scores indicate higher perceived physician empathy. The reliability of the scale was high (Cronbach’s alpha=.94).

We measured distress before and after the consultation (T2 and T4) using the 6-item state scale Spielberger State-Trait Anxiety Inventory (STAI-6) (1= “not at all”- 4=“very much so”) (T2 and T4).^38, 39^ This inventory assesses a psychophysiological state following the perception of threat. Reliability was good (Cronbach’s alpha=.77). Change scores in distress were calculated and used in further analyses by subtracting STAI-6 scores before (T2) from STAI-6 scores after the consultation (T4).

Patients’ level of social anxiety was assessed using a commonly used screening tool, the Short Form of Social Interaction Anxiety Scale (SIAS-6) and Social Phobia Scale (SPS-6) (T1). It assesses general social interaction anxiety (e.g., talking to strangers) and fear of being observed (e.g., eating in the presence of others), respectively, and has 12 items (0= “not at all true of me”–5=“extremely true of me”).^40, 41^ High scores on either of the two scales indicate social anxiety. Reliability was high (Cronbach’s alpha=.84).

### Data Preparation

The eye-tracking data was analyzed with software to automatically create areas-of-interest around faces, created for this study based on a previously developed algorithm.^32^ The software was used to create an area-of-interest around the faces of all individuals present in the videos. The output of the software indicated for each video frame (40ms) whether the gaze of the physician was focused on the face of the patient or the caregiver (if present), i.e., within the area-of-interest (coded as “1”) or not (“0”). This output was used to compute the average dwell time of face gaze, which is measured as the mean duration of each gaze instance towards the face. For more details, see Jongerius et al..^32^

### Statistical Analysis

A required sample size of 96 patients was calculated based on multilevel regression analysis (80% power, *α*=.05, medium effect size=.25), which yields a sample size of *N*=53.^42, 43^ To account for the clustering of consultations within physicians, we estimated a design effect of 1.8 (Deff=1+(*m*-1)ICC), where *m* is the number of consultations per physician=5 and ICC=.2.^44^ In total, this yielded a required *N* of 53×1.8=95.4. Descriptive analyses were done using SPSS 26.^45^ Because of the nested data, we conducted a multilevel analysis with face gaze (average dwell time) as an independent variable and patients’ reported trust in physicians as a dependent variable. We applied a multilevel Tobit model because trust showed a left-skewed distribution with a ceiling effect.^46^ In addition to the crude analyses, we adjusted for gender, age, educational level, caregiver presence, and social anxiety because these variables could influence either the level of face gaze or trust.^47, 48^ To test if social anxiety moderated the effect of face gaze on trust, we examined the interaction between social anxiety and face gaze. We repeated these multilevel models twice, using the exploratory-dependent variables^1^ perception of physician empathy and^2^ distress instead. All multilevel analyses were performed with STATA/SE 14.^49^ All test outcomes were considered significant with an alpha of *p<*.0.05.

## Results

### Descriptives

In total, 16 physicians participated, with a median age of 33.5 years (ranged 29 to 38 years). They had a median of 4.5 years in training (ranged from 2 to 6 years). Physicians consulted with between 2 and 14 patients (median=6). In total, 202 patients were approached for participation, of whom 130 telephonically agreed to participate. Reasons for declining to participate were “not interested” or “will cancel appointment for other reasons” or “did not speak Dutch.” We collected 102 measurements of which 100 eye-tracking registrations were complete. Patients were on average 58.1 years old (SD = 14.0). Physical examination was performed in 24 consultations (24%) and a caregiver was present in 17 consultations (17%). Consultations (disregarding physical examinations) had a median duration of 14.1 min (range=3.0–45.1). Table 1 provides an overview of the sample characteristics. Table 2 provides the outcomes of our measures.

**Table 1:** Sociodemographic Characteristics of Patients and Physicians

	n (%)
**Patients (*N*=100)**	
Female gender	47 (47%)
Self-identified nationality	
Dutch	94 (94%)
Other European nationalities	2 (2%)
South American	3 (3%)
Middle East	1 (1%)
Education level (*n*=99)	
None/primary school	29 (29%)
Secondary/lower level vocational school	50 (50%)
College/university	20 (20%)
**Physicians (*N*=16)**	
Female gender	8 (50%)
Self-identified nationality	
Dutch	15 (94%)
Arabic	1 (6%)

**Table 2:** Outcomes of Independent, Dependent, Moderator and Exploratory Variables

	n	Median	Range
Independent variable			
Face gaze (average dwell time) in seconds	100	1.0	0.1–6.8
Dependent variables			
Trust (Wake Forest Physician Trust Scale, range=1–5)	100	4.6	2.1–5
Perceived empathy (Consultation And Relational Empathy, range=10–50)	93	43.9	27–50
Change in distress (Spielberger State-Trait Anxiety Inventory-S)	100	-2.33	−23.33 to 23.33
Moderating variable			
Social anxiety (Social Phobia Scale-6 and Social Interactional Anxiety Scale-6, range=0–40)	99	1.0	0–20

*Note:* For the empathy measure we omitted data of all patients who filled in “does not apply” on at least 3 items (*n*=6; 6%), which is an acceptable number for this measure.^36^ One patient did not respond to the social anxiety questionnaire. Therefore, data of this patient were excluded from moderation analyses

### Outcomes of the Multilevel Associations

#### Association between Physician Face Gaze and Patients’ Trust

Face gaze and trust were not significantly associated as indicated by our multilevel regression. The regression coefficient of −0.16 (*P*=0.098) corresponds with a small to moderate effect size (*D*=−0.22) (Table 3).^44^ When adjusting for gender, age, education, caregiver presence, and social anxiety, there was a significant, inverse relation between face gaze and trust. The regression coefficient of −0.17 (*P*=0.048) corresponds with a small to moderate effect size (*D*=−0.24). Social anxiety did not moderate the relation between face gaze and trust (*P*=0.32). The addition of gender, age, education level, and caregiver presence as covariates did not alter test results (*P*=0.48).

**Table 3:** Multilevel Regression Models between Physician Face Gaze and Patient Trust, Perceived Empathy, and Distress

	Trust Wake Forest Physician Trust Scale			Perceived empathy Consultation and Relational Empathy			Distress Spielberger State-Trait Anxiety Inventory-S		
	*Β* (95%CI)	*P* (n)	*D*	*Β* (95%CI)	*P* (n)	*D*	*Β* (95%CI)	*P* (n)	*D*
Crude	−0.16 (−0.34, 0.03)	0.098 (100)	−0.22	0.90 (−2.56, 4.37)	0.609 (93)	0.08	1.37 (0.52, 3.27)	0.155 (100)	0.14
Adjusted for patient gender, age, education, social anxiety, and caregiver presence)	−0.17(−0.34, −0.00)	0.048 (99)	−0.24	2.22 (−1.15, 5.60)	0.197 (93)	0.21	1.34 (−0.58, 3.27)	0.172 (100)	0.13

*Note:* all models had physician as a nesting factor. Crude analysis is the multilevel regression between the outcome (trust, perceived empathy, distress) and face gaze. Models are adjusted for patient, gender, age, education, social anxiety, and caregiver presence. All intercepts (*B*), confidence intervals (CI), values of significance (*P*), sample size (*n*), and effect sizes (*D*), for each model are displayed

#### Association between Physician Face Gaze and Exploratory Outcome Measures

There was no significant relation between face gaze and perceived empathy as indicated by our multilevel regression. The regression coefficient of 0.90 (*P*=0.609) corresponds to a small effect size (*D*=0.08). Adjustment for gender, age, education level, caregiver presence, and social anxiety did not alter test results (*P*=0.197) and corresponded with a small to moderate effect size (*D*=0.21). There was no significant relation between face gaze and change in distress as indicated by our multilevel regression. The regression coefficient of 1.37 (*P*=0.155) corresponds with a small effect size (*D*=0.14). The addition of gender, age, education level, caregiver presence, and social anxiety as covariates did not change this result.

## DISCUSSION

We investigated whether physicians’ face gaze towards their patients predicted patients’ trust. Our results suggest that more face gaze is associated with lower trust, but only when correcting for patient gender, age, education level, and social anxiety, and for the presence of a caregiver. No relation was found between physician face gaze and patient perception of physician empathy or patient distress. Moreover, patients’ social anxiety did not moderate the relation between physician face gaze and patient trust levels.

We hypothesized a positive relation between physicians’ gaze towards the patient and patients’ trust,^5, 11^ but found the opposite. This contrast may be explained by methodological differences between our study and previous research. We were the first to measure face gaze with eye-tracking in this setting, which enables high observational precision.^2^ Previous research assessed face gaze using less objective methodologies, such as observer-based coding of video recordings.^7–9, 21^ Perhaps, researchers are biased to believe that face gaze has positive effects, which may have influenced their assessment and hence the results. It is possible that face gaze has subconscious effects that are yet poorly understood. To the best of our knowledge, only one previous study using more traditional observational methods found a negative relation between physician face gaze and a patient outcomes, specifically distress.^22^ In the current study, this specific relation was not confirmed. This could be due to differences in patient characteristics: the previous study included patients visiting for genetic breast cancer counseling, whereas our study included internal medicine patients. Patients visiting for genetic breast cancer counseling, often do so to receive emotional support or reduce worries, which differs from follow-up care of the internal medicine patients.^22^

Another possible explanation for a negative relation between face gaze and trust could lie in the physician’s perception of the quality of communication.^50^ Trust is a dyadic construct that is created and adjusted in interaction.^50^ Longer face gaze duration in the current study may occur in more difficult conversations, e.g., involving interactions about emotionally charged issues or in interactions, in which the physician senses a lack of trust in the patient.^22^ In those circumstances, the physician may unconsciously pay more attention and gaze more extensively to the patient. At the same time, this could feel less comfortable for the patient and may lead to less positive judgements about the physician. Unfortunately, we could not test this assumption because we did not classify the specific content or quality of the communication. Future research should take into account the content of the consultation to explain the relation between face gaze and outcomes.

The negative relation between face gaze and trust could alternatively be explained by the “eye contact effect”,^11^ meaning that perceived gaze affects neurobehavioral responses and cognitive processing.^51, 52^ These responses to gaze include, for example, higher bodily self-awareness and a decreased capability to perform cognitive tasks.^52–56^ In our setting, this would mean that the feeling of being observed could interfere with patients’ trust in their physician. A consultation with a physician can evoke stress in the patient which, in combination with higher levels of physician gaze and associated neurobehavioral and cognitive effects, could lead to decreased trust in the physician because the patient feels overly observed by the physician.^57^ Previous studies demonstrating the “eye contact effect” were experimental.^52–56^ To further investigate whether the “eye contact effect” may be relevant to the physician-patient setting, we recommend the integration of such measurements (e.g., assessing autonomic responses) in clinical studies.

Social anxiety did not moderate the relation between physicians’ face gaze and patient trust. Previous studies found such associations in student samples (i.e., including young and highly educated people) and used different questionnaires to assess social anxiety.^23, 24^ In contrast, our sample included more variation in age and education levels. Furthermore, social anxiety levels were low in our sample, with little variation, which could explain the lack of a moderation effect. We recommend that future research further entangles the relation between face gaze and trust, especially for samples of socially anxious individuals. This could be relevant to understand and adapt to the communication needs of such socially more vulnerable individuals.

A limitation of this study is that we did not distinguish between face gaze towards the patient versus towards the caregiver. Therefore, the measured gaze towards the patient could be an overestimation in the minority of consultations including a caregiver. Secondly, the effects of face gaze of the physician towards the patient on trust were only present when adjusted for confounding variables, i.e., gender, age, educational level, the presence of a caregiver, and social anxiety. Third, we cannot exclude the possibility of a Hawthorne effect, meaning that results may have been biased because physicians were aware of being observed.^58^ Finally, we did not take into account the face gaze of the patient towards the physician, because we did not want to overly burden patients. Earlier studies show that the level of face gaze in a conversation depends on both individuals.^59^ Face gaze is a bidirectional, interactional phenomenon, in which individuals adjust their level of face gaze towards each other. Future studies should therefore additionally take into account patients’ face gaze towards the physician. Furthermore, our sample was largely homogeneous. Future research could take into account cultural context, such as ethnicity, of patients and physicians.^60^

Study strengths include being the first to utilize mobile eye-tracking glasses, which allow for more objective and precise measurement of physician face gaze compared to other modalities (e.g., observer-based coding of video recordings).^2^ Second, we have included a much larger sample compared to earlier mobile eye-tracking studies.^24, 61–63^

Concluding, we unexpectedly found physician face gaze to be negatively associated with patients’ trust in their physician. Therefore, our results challenge the current view that physician gaze is by definition beneficial to patients and their trust, encouraging continued and more in-depth research on this topic. The results give strength to the nuance of communication skills, in that good communication skills are not simply a discrete set of behaviors, but rather, a set of behaviors that must be appropriate for certain contexts and situations. Ultimately, these findings may lead to enhancement of physician-patient communication, improving their relation and thereby the quality of care. We found no relation between face gaze and other outcomes, i.e., patients’ perception of physician empathy and patient distress. Future studies should be performed to better understand the relation between nonverbal communication and outcomes. These should involve the bidirectional study of face gaze in physician-patient consultations and account for the level of emotional talk in the consultation.
